# A Woman's Experience in a London General Hospital

**Published:** 1913-11-01

**Authors:** 


### HOSPITALS FROM THE PATIENTS' POINT OF VIEW.

**(Contributions to this Section are Invited.)**

### A Woman's Experience in a London General Hospital.

My experience of hospital life was a short one, but it has impressed itself very deeply on my mind on account of its having been quite unfamiliar to me up to that time. I had cut my hand badly, and it became necessary to have a tendon united in order to regain the use of a finger. Apart from the local injury, I was feeling quite well, and it came as an unpleasant surprise to learn from the surgeon that the operation would take some time, and that I must be admitted to the wards. However, I attended the hospital on the day named, and after waiting for over two hours was informed that there was no vacancy for me, and that I was to attend again on that day week. I did so, and after waiting three hours was appointed to a ward, and was taken there by lift with another patient. On each occasion I had to reach the hospital before 10 a.m., and, although I got very hungry as time went on, I did not notice any place for refreshments (although I found afterwards that there was such a place in another part of the Out-Patient Department). Having reached the ward, I was told that my bed was not ready, and I was given some beef-tea and bread. I sat at a table in the ward all the afternoon, and about three o'clock I was given some very washy tea (not worthy of the name) in a mug with no handle and no saucer. After that I was taken to the bathroom and left to bath myself, a probationer nurse coming later on to wash and comb my hair, though it was very obvious that this was a quite unnecessary precaution. As she did not properly wash the soap out, the process did not add to my comfort. At about 7 o'clock I was told that my bed was ready, and I undressed by the side of it without any screen round me. We had prayers at 8 o'clock, and were then supposed to settle down for the night. The sister, however, was not too strict, and she would sometimes allow special visitors in during the early part of the evening.

I cannot say that I had a proper night's sleep during any of the ten days I was in hospital. For one thing, the fact that we had nothing to eat after supper at 7 o'clock was very disturbing to anyone accustomed to a meal later in the evening. For another thing, my bed was close to the ward table, by which the doctors would always stand to gossip with the nurses during their last round, often at a comparatively late hour. Then, later still, the night sister would come in, and however quiet she were, her various comings and goings were distracting. When at last I did get to sleep, directly after (it seemed) I was awakened for five o'clock breakfast. The early hour of this meal is a common complaint amongst hospital patients, and while making every allowance for the amount of work to be got through by the staff, it does appear strange that sick people have to do what would be eminently disagreeable and distasteful to them if they were well. With regard to the food, I found the dinner nearly always good, and naturally looked to it to supply my chief nourishment. The other meals were poor, the tea being always sloppy (due almost entirely I believe to its being badly made) and the bread and butter badly and unappetisingly prepared. We could have our own eggs for tea, and were sometimes given bread and dripping instead of bread and butter for lunch. At dinner time, in the way of "afters," there was eternally rice pudding; but once or twice, as a special favour, I was given stewed gooseberries, without, however, the slightest trace of sugar in them or on them. I have since been informed that I should have asked for some of this, but on my first day I had asked a nurse if I might go to the lavatory, and she had refused so crossly and tartly that I never asked for anything again. So far as the sisters were concerned, both day sister and night sister were very kind to me, as was also the resident medical officer; but the house-surgeon I was under was a prig, who stood in an affected and apish way talking to the nurses whenever he came in the ward. When I had had my operation and he was dressing the hand, he nearly jumped down my throat because I rested it on the bed for a moment—after which he went on winding the bandage round, looking at and talking to a nurse all the time.

My operation passed off well (after having been postponed for some days), and as soon after as possible I left the hospital. If my recollections of it are not as pleasant as I should have liked them to be, perhaps it is because my general good health rendered me more critical, and the constraint more irksome. But even allowing for that, I cannot help thinking that the treatment of the sick is sometimes too "professional," and that not enough allowance is always made for the human element.

